# Development and content validation of an instrument to assess the prevalence of psychiatric medication use among dental students at Brazilian universities

**DOI:** 10.1186/s12909-025-07843-y

**Published:** 2025-09-10

**Authors:** Aléxia Caroline Leandro da Conceição, João Victor Frazão Câmara, Ana Flávia Almeida Barbosa, Ane Poly, Gisele Damiana da Silveira Pereira

**Affiliations:** 1 School of Dentistry, Federal University of Rio de Janeiro, Rio de Janeiro, RJ Brazil; 2 Clinic of Operative Dentistry, Periodontology and Preventive Dentistry, Saarland University, Homburg Saar, Germany; 3 School of Dentistry, State University of Rio de Janeiro, Rio de Janeiro, RJ Brazil; 4 College of Dentistry, University of Florida, Gainesville, FL USA

**Keywords:** Dental students, Validation study, Surveys and questionnaires, Mental disorders, Anxiety

## Abstract

To develop and validate a measurement instrument aimed at assessing the prevalence of psychiatric medication use among final-year dental students enrolled in Brazilian universities. Methods: The process emphasized both the construction of the questionnaire and the collection of evidence for its content validity. A mixed-methods approach was employed, integrating quantitative and qualitative methodologies. The initial stages involved defining the main research problem, establishing the instrument’s objectives, determining its conceptual domains, identifying the target population, and constructing the items, scoring system, and response scales. Based on a comprehensive literature review, a preliminary questionnaire consisting of 23 open- and closed-ended items was created. This version was submitted to a panel of eight expert judges—academics and/or researchers specializing in Psychology, Psychiatry, Pedagogy, and Dentistry/Instrument Development—tasked with evaluating each item’s clarity. Results: Content analysis was conducted using consensus thresholds. Quantitatively, the agreement rate among judges was calculated as the proportion of experts endorsing each item. Items with less than 90% agreement were revised; items with 90% or greater agreement were considered acceptable. In the first round of evaluation, 14 items were rejected. After modification based on judge feedback, all items were approved in the second round. The high level of agreement among experts was deemed sufficient to provide evidence of content validity. Following expert validation, a pre-test was conducted with dental students’ representative of the target population. Based on both qualitative and quantitative feedback, two questions were adjusted, leading to the development of the final version of the instrument. Conclusions: These initial findings and methodological steps form the foundation for the instrument’s definitive application in future research assessing psychiatric medication use among undergraduate dental students.

**Supplementary Information:**

The online version contains supplementary material available at 10.1186/s12909-025-07843-y.

## Introduction

Mental disorder (MD) is defined as a significant disturbance in an individual’s cognition, emotional regulation, or behavior, and is often associated with an inability to perform social, occupational, or other important activities. This reflects a dysfunction in psychological, biological, or developmental processes [1]. Its etiology should be understood as the result of an interaction between an individual’s psychological interpretation capacity and their bodily perceptions—whether physiological or pathological. Mental disorders can emerge in any individual, at any stage of life, as stress and everyday events may act as triggers for the development of such conditions [2, 3]. 

With a significant increase in cases in recent years, mental disorders have been described as one of the main global public health challenges in this century [4, 5]. In 2022, the World Health Organization (WHO) released the World Mental Health Report, estimating that in 2019, approximately 970 million people worldwide were living with mental disorders, 15.6% of whom were from the America [5]. In Brazil, prevalence rates range from 17 to 35% of the population, with some estimates reaching as high as 50% [6]. Nunes et al. (2016) reported that 26.8% of Brazilian adults exhibited at least one symptom of a psychiatric comorbidity [7]. 

Among the most prevalent mental disorders in society are depression, anxiety disorders, developmental disorders, bipolar disorder, and attention-deficit/hyperactivity disorder (ADHD) [5, 8–12]. In connection with these conditions, psychological and somatic distress, social isolation, and low occupational and academic performance are commonly reported consequences [7]. 

The incidence of these disorders is strongly correlated with the increasing use of psychiatric medications, which are prescribed to manage or reverse the adverse effects caused by neurological imbalances associated with mental illness [13, 14]. This rise in medication use raises concerns regarding indiscriminate consumption, non-prescription use, and users’ lack of awareness about potential side effects and associated risks [15, 16]. 

Regarding university students and mental health, the literature indicates that varying levels of stress and psychological exhaustion are frequently observed during undergraduate studies [17]. A World Health Organization (WHO) survey conducted in 21 countries revealed that 20.3% of undergraduate students experienced mental health problems [18], with higher prevalence reported among health-related programs, where approximately 15–29% of students were found to suffer from some form of psychiatric disorder [4]. University students’ mental well-being has been associated with multiple factors, including monthly household income, ethnicity, social life, parental education and occupation, general interests, gender, age, academic performance, pressure to succeed, and postgraduate plans [19]. 

Among dental students specifically, symptoms such as somatic complaints, anxiety, depression, stress, burnout, and even suicide risk have been identified [20, 21]. Studies conducted with healthcare professionals have demonstrated a link between mental health issues, particularly depression, stress, and burnout, and reduced quality of professional care and lower patient satisfaction. These findings underscore the importance of investigating mental health among healthcare providers, given the potential impact on the quality of care delivered [22, 23]. 

The demanding academic routine, extensive workload, the need to develop technical skills, and increasing responsibilities may contribute to the rising consumption of psychiatric medications and other psychoactive substances among dental students [24]. The proportion of undergraduates using psychiatric drugs has increased over the past decade [25]. It is important to emphasize, however, that the use of such substances can produce varying effects depending on dosage and drug interactions [24]. 

Moreover, the indiscriminate use of psychiatric medications poses a significant public health concern, as these substances can lead to dependency and adverse effects such as behavioral changes, cognitive impairment, drowsiness, appetite fluctuations, and disturbances in motor and autonomic functions [24]. A literature review was conducted using key descriptors to identify the existence of studies related to the topic. The search revealed the absence of validated instruments addressing the proposed theme. Thus, the objectives of this study were to develop a measurement instrument to assess the prevalence of psychiatric medication use among dental students regularly enrolled in the final year of undergraduate programs at Brazilian universities and to assess whether these students perceive unwanted effects from medication use and whether these perceived effects have an impact on their clinical care. With a focus on the questionnaire development process and evidence of its content validity.

## Materials and methods

### Ethical aspects

The research project was approved by the Research Ethics Committee of the School of Dentistry at the Federal University of Rio de Janeiro (CEP/FO/UFRJ), CAAE: 66900923.6.0000.0268, number 5.947.346. The study adhered to the resolutions of the National Health Council (CNS) numbers 466/2012 and 510/2016, as well as the guidelines outlined in Circular Letter number 1/2021 from the National Research Ethics Commission (CONEP), which address procedures for research involving any stage conducted in a virtual environment.

### Research design

This study involved the construction and validation of a mixed-format questionnaire (including both open- and closed-ended questions), designed as an opinion survey to be employed in a quantitative, observational, cross-sectional, and descriptive web-based study. The questionnaire will be self-administered and virtually distributed through academic networks, institutional groups, e-mail, and messaging applications across the five regions of Brazil. The research will target final-year undergraduate dental students from both public and private Brazilian dental schools. Sampling will be non-probabilistic for convenience. The methodology followed a rigorous instrument development process for data collection. Fundamental methods and stages were adhered to during the construction of the items composing the final evaluation instrument [26]. 

### Establishment of the conceptual framework, instrument objectives, and target population

This stage was responsible for defining the context of the instrument to guide the subsequent development of the research domains and items. The research instrument must be appropriate to the investigative question, and this principle should guide the entire research development process [27]. 

After establishing a link between the objective and the concepts addressed, the target population was defined. Defining the target population for a measurement instrument aims to determine how its applicability can be planned and the extent to which the instrument can be understood by its users [26]. The target population was defined as dental undergraduate students regularly enrolled in the final year at public and private universities in Brazil, without restrictions on age, gender, ethnicity, or socioeconomic status, who electronically accept the Informed Consent Form (ICF). Accordingly, before responding to any research questions, participants must click “I have read, understood, and consent to participate in this research” to be redirected to the questionnaire.

The inclusion criteria for participation invitation were: regularly enrolled in a Dentistry program in Brazil; being in the final year of the Dentistry undergraduate course and having internet access to complete the survey virtually. On the other hand, the exclusion criteria were: students who have suspended their enrollment or have abandoned the Dentistry course; students enrolled in years other than the final year of the course and students without internet access.

### Item construction and organization, and instrument structuring

The study instrument was developed through a literature review using the following key descriptors: “Dental Students,” “Validation Study,” “Psychotropic Drugs,” “Surveys and Questionnaires,” “Mental Disorders,” “Anxiety,” and “Depression.” These descriptors provided the basis for aligning each question with the main objective of the instrument, as well as for constructing the response items, in accordance with the definitions and objectives established in the initial phase [26]. 

The literature search was conducted in PubMed, BVS, Embase, Scopus, Web of Science, and Cochrane databases. The focus was placed on national and international studies, available in English and Portuguese, that used questionnaires with undergraduate students and were published within the last 10 years. Literature-based evidence and database searches are commonly used in the development of measurement instruments [28]. 

Following the literature review, three research domains were defined: Psychotropic Use, Student Health, and Mental Health. These domains guided the construction of a variety of items aligned with the underlying constructs, contributing to the quality and robustness of the instrument’s content, which would later undergo validation. The questionnaire was then organized into thematic blocks according to the topics addressed [29]. The items were developed based on the predefined criteria [26]. 

The final questionnaire consisted of twenty-seven mixed-format questions designed as an opinion survey. It was structured into blocks, including sociodemographic questions (family income, age, ethnicity, and gender); questions related to medication use (name of the drug, duration of use, and observed effects); and questions addressing the participant’s self-perception regarding the potential impact—if any—on clinical practice.

### Selection of the scientific expert committee

Eight specialists in Psychology, Psychiatry, Pedagogy, and Dentistry/Psychometrics, Instrument Adaptation and Development—two from each field—were invited via an email invitation letter to participate in the study. All invited experts accepted the invitation to take part in the research. Eligibility criteria for inclusion in the expert committee included professional experience and expertise in the research topic and/or in the development and validation of research instruments. The selected experts were professors and/or researchers with recognized proficiency in instrument validation, members of academic or professional councils in their field, and/or certified specialists. Only individuals meeting at least two of these criteria were invited to join the committee.

### Evaluation and structuring of the questionnaire according to the scientific experts’ suggestions

The instrument was tested regarding the representativeness and/or adequate coverage of the selected items in relation to the objectives of the construct [26]. The questionnaire was sent electronically to the panel of experts. The specialists were instructed to evaluate each item of the instrument and to provide comments, suggestions, and modifications based on criteria such as objectivity, clarity of item wording, item representativeness, interpretability of content, sequence of items, and the applicability of the developed instrument. The aim was to eliminate any incomprehensible items and ensure that all terms were addressed in a way that maintained the respondent’s interest throughout the entire questionnaire [26]. For item representativeness/relevance, each question was assessed to determine whether it truly reflected the intended concepts, whether it was relevant, and whether it was appropriate for achieving the proposed objectives. The available response options were: 1 – Not representative; 2 – Requires major revision to be representative; 3 – Requires minor revision to be representative and 4 – Representative. For item clarity, each question was evaluated to assess whether its wording made the concept understandable and whether it clearly expressed what it aimed to measure. After asking whether the item was clear and comprehensible, evaluators responded with “Agree” or “Disagree” and scored the item using: 1 - Not clear; 2 - Slightly clear; 3 - Quite clear and 4 - Very clear.

Open text fields were also provided so that the judges could suggest improvements to the item, propose additions or eliminations, or offer general comments. In this way, the panel assessed whether each concept was adequately covered and whether all relevant dimensions were included. Subsequently, a quantitative and qualitative review was conducted, taking into account the experts’ suggestions, which were discussed to ensure the necessary adjustments were made. The evaluation was based on the percentage of agreement (%) from the expert committee, calculated by the ratio between the number of consultants who agreed and the total number of consultants. An agreement rate below 90% triggered discussion and revision of the item, while a rate of 90% or higher indicated the item was considered adequate [26, 30]. 

Furthermore, the Content Validity Index (CVI) was calculated to validate each item, using the ratio between the sum of scores “3” and “4” and the total number of responses. Items receiving scores of “1” or “2” were revised or removed when necessary. To validate the instrument, a minimum agreement threshold of 0.80 was required [30]. In addition, to assess the extent to which the agreement between the evaluators, item by item, went beyond the agreement that could occur by chance alone, we used the Polit’s kappa modified (PMK). Items with kappa (k) < 0.74 were considered inadequate and rewritten [31]. 

Once this step was completed, the revised questions were resent to the committee, and the evaluations were repeated. After final analysis and approval of all items, the resulting version of the questionnaire was subjected to pre-testing with the target audience, who received a remotely generated form through the Google Forms platform containing the updated version of the instrument.

### Pilot testing of the instrument in the target population and finalization of the questionnaire based on pre-test feedback

The pre-test was conducted with the objective of verifying whether all items were understandable to the target population for whom the instrument was designed, thereby avoiding potential issues during the final application phase. For this purpose, 20 undergraduate dental students were randomly selected to validate the questionnaire’s reliability, assess their level of understanding of the questions, and record the average response time [32]. The data collection instrument was distributed via email and the WhatsApp application. The average time required to complete the questionnaire was approximately 6 min. Following the questionnaire items, participants were asked whether they found the questions intelligible, with the following closed-ended response options: (a) Fully understood; (b) Partially understood; and (c) Did not fully understand. After this question, participants were provided with an open-ended space to offer suggestions in case a particular item was not fully understood or if they believed any modifications were necessary.

### Finalization of the questionnaire

After analyzing and considering the feedback received, the questionnaire items were refined. Of the 23 questions included in the instrument submitted for pre-testing, two were modified based on suggestions from the pre-test group. Based on the pre-test performance, the final version of the instrument was drafted (Supplemental material). Participant recruitment for the study will be conducted via email, through which both the invitation and the questionnaire will be sent.

The final questionnaire was structured using the Google Forms platform (Google, California, United States), configured not to collect any identifiable information, such as names, email addresses, IP addresses, or any other personal data. Additionally, due to the sensitive nature of some questions, the informed consent form, approved by the ethics committee, states that participants have the right to withdraw from the study at any time if they feel uncomfortable. Agreement to this form is mandatory before proceeding to the questionnaire.

**Fig. 1:**
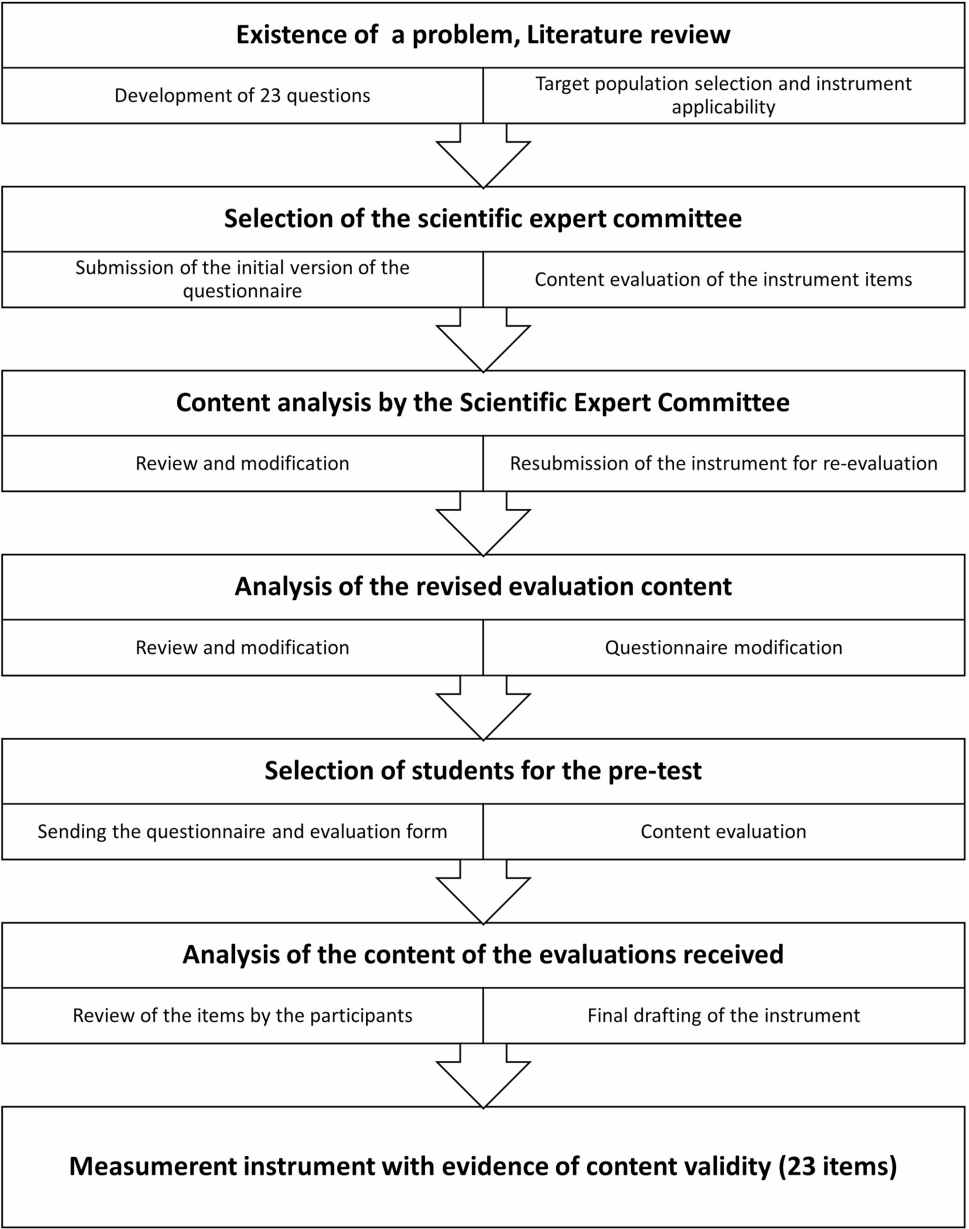
Flowchart detailing the study design

The finalized version consisted of 23 questions: (a) 10 sociodemographic questions (e.g., family income, age, ethnicity, gender); (b) 11 questions related to the use of psychiatric medication (e.g., drug name, duration of use, observed effects) and (c) 2 questions addressing participants’ self-perception regarding potential interference of medication use in clinical practice (Fig. 1).

### Sample size calculation for instrument application

According to data extracted from the National Registry of Higher Education Courses and Institutions, regulated by Normative Ordinance number 21, dated December 21, 2017, there are currently 553 active undergraduate dentistry courses throughout Brazil, comprising 488 private institutions and 65 public institutions (http://emec.mec.gov.br/). Considering that most dentistry courses last eight semesters and approximately 75.117 seats are offered each semester, it is estimated that there are around 150.234 students enrolled in the final year of the dentistry program nationwide. For sample size calculation, the software G*Power 3.1.9.2 (Heinrich Heine University Düsseldorf, Germany) was used, considering a precision of 50%, a confidence interval of 95%, and a margin of error of 4%. Based on these parameters, a minimum sample size of 598 respondents was determined to ensure representativeness and reliability of the collected data.

## Results

### Committee of expert judges

Each questionnaire item was evaluated using the Content Validity Index (CVI), requiring a minimum score of 0.80 for approval, by the percentage of agreement, which required a rate equal to or greater than 90% for acceptance, and by Polit’s kappa modified (PMK), which demanded a value greater than 0.74 for approval. Table 1 presents the values obtained during the evaluation conducted by the committee of expert judges. Regarding the Content Validity Index (CVI) and the PMK, the introductory text and items 8, 9, 22, 23.1, 23.2, 23.3, and 23.4 obtained values below 0.80 and 0,74, respectively, in the clarity criterion. Additionally, in terms of representativeness, items 4 and 10 scored below 0.80 on the CVI and lower than 0,74 on the PMK. Regarding the percentage of agreement, the introductory text and items 1, 2, 4, 7, 8, 13, 22, 23.1, 23.2, and 23.3 received ratings below 90%.

**Table 1:** Evaluation by the scientific committee of judges

Item	Content validity index (CVI)		Polits’s modified kappa		Percentage of agreement (%)
	Clarity	Represen-tativeness	Clarity	Represen-tativeness	
Title	1.00	1.00	1,00	1,00	100.0
Introductory text	0.75*	1.00	0,72*	1,00	75.0*
Question 1	1.00	0.87	1,00	0,87	87.5*
Question 2	0.87	1.00	0,87	1,00	87.5*
Question 3	1.00	1.00	1,00	1,00	100.0
Question 4	0,87	0.75*	0,87	0,72*	75.0*
Question 5	1.00	1.00	1,00	1,00	100.0
Question 6	1.00	1.00	1,00	1,00	100.0
Question 7	0.87	1.00	0,87	1,00	87.5*
Question 8	0.50*	1.00	0,31*	1,00	75.0*
Question 9	0.75*	1.00	0,72*	1,00	100.0
Question 10	0.87	0.75*	0,87	0,72*	100.0
Informative text	0.87	1.00	0,87	1,00	100.0
Question 11	0.87	1.00	0,87	1,00	100.0
Question 12	0.87	1.00	0,87	1,00	100.0
Question 13	0.87	1.00	0,87	1,00	87.5*
Question 14	1.00	1.00	1,00	1,00	100.0
Question 15	0.87	1.00	0,87	1,00	100.0
Question 16	0.87	1.00	0,87	1,00	100.0
Question 17	1.00	1.00	1,00	1,00	100.0
Question 18	0.87	0.87	0,87	0,87	100.0
Question 19	1.00	1.00	1,00	1,00	100.0
Question 20	0.87	1.00	0,87	1,00	100.0
Question 21	1.00	1.00	1,00	1,00	100.0
Question 22	0.75*	1.00	0,72*	1,00	75.0*
Question 23.1	0.75*	0.87	0,72*	0,87	87.5*
Question 23.2	0.75*	0.87	0,72*	0,87	75.0*
Question 23.3	0.75*	0.87	0,72*	0,87	75.0*
Question 23.4	0.75*	1.00	0,72*	1,00	100.0
Question 23.5	1.00	1.00	1,00	1,00	100.0

*Value below the threshold required for item approval

Following the discussion of the judges’ suggestions, the rejected items (introductory text, 1, 2, 4, 7, 8, 9, 10, 13, 22, 23.1, 23.2, 23.3, and 23.4) were revised and resubmitted for reevaluation. After analyzing the new data, all revised items achieved a CVI and PMK of 1.00 for both clarity and representativeness, as well as a 100% agreement rate (Table 2).

**Table 2:** Re-evaluation by the scientific committee of judges

Item	Content validity index		Polits’s modified kappa		Percentage of agreement (%)
	Clarity	Represen-tativeness	Clarity	Represen-tativeness	
Introductory text	1.00	1.00	1.00	1.00	100.00
Question 1	1.00	1.00	1.00	1.00	100.00
Question 2	1.00	1.00	1.00	1.00	100.00
Question 4	1.00	1.00	1.00	1.00	100.00
Question 7	1.00	1.00	1.00	1.00	100.00
Question 8	1.00	1.00	1.00	1.00	100.00
Question 9	1.00	1.00	1.00	1.00	100.00
Question 10	1.00	1.00	1.00	1.00	100.00
Question 13	1.00	1.00	1.00	1.00	100.00
Question 22	1.00	1.00	1.00	1.00	100.00
Question 23.1	1.00	1.00	1.00	1.00	100.00
Question 23.2	1.00	1.00	1.00	1.00	100.00
Question 23.3	1.00	1.00	1.00	1.00	100.00
Question 23.4	1.00	1.00	1.00	1.00	100.00

### Pre-test results

**Fig. 2:**
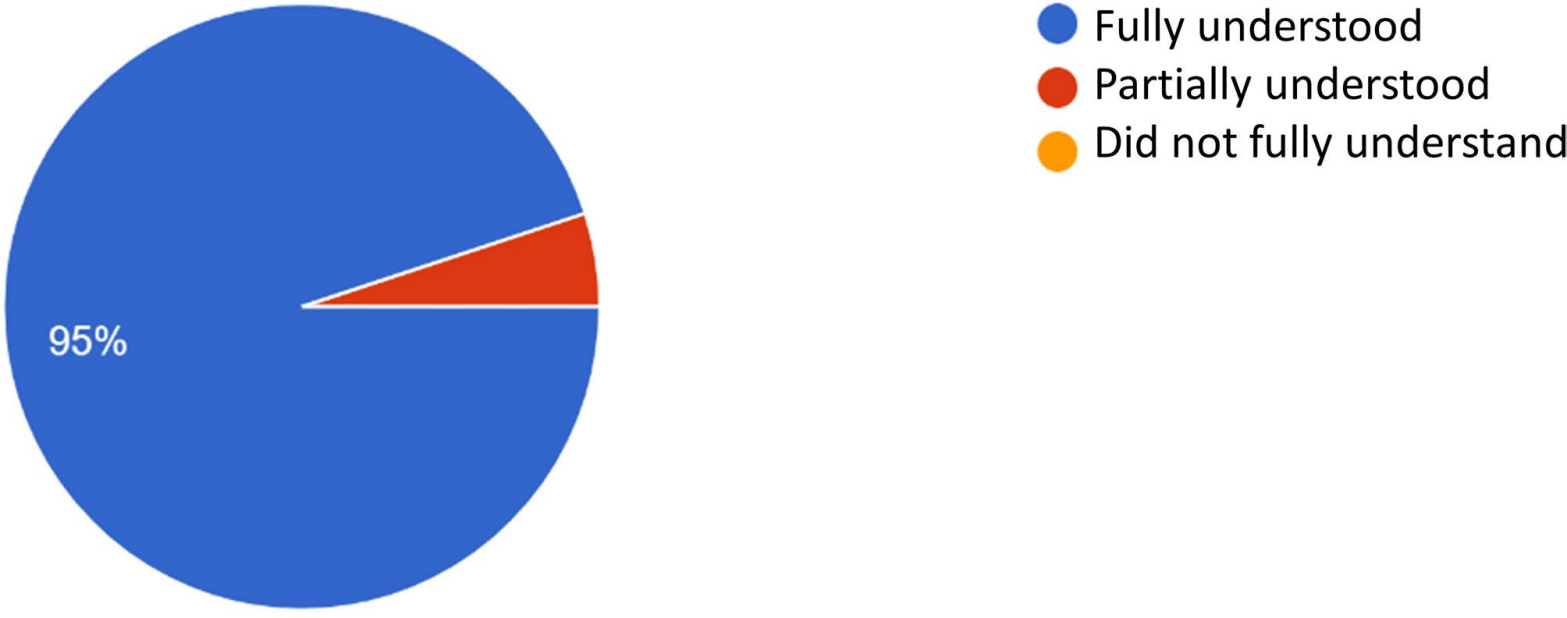
Evaluation of items question 4, question 20 and question 22

Twenty penultimate-year dental students participated in the pre-test. Each participant completed an online questionnaire created using the Google Forms platform. All twenty participants (100%) reported that they fully understood the majority of the instrument’s items, without leaving any comments or suggestions in the comment section, with the exception of items 4, 9, 12, 20, and 22. Nineteen participants (95%) indicated that they fully understood items 4, 20, and 22, while one participant (5%) reported partially understanding these items. Items 9 and 12 received 100% (*n* = 20) of the responses as “fully understood.” However, these items did receive comments. All comments were carefully considered, discussed among the researchers, and items 9 and 20 were adjusted based on the suggestions provided by the pre-test participants (Fig. 2).

## Discussion

During the literature review, no existing questionnaire was found that comprehensively addressed the research objective. Therefore, it was necessary to develop a study instrument capable of assessing the prevalence of psychiatric medication use among dental students enrolled in Brazilian universities. Additionally, the instrument aimed to evaluate whether dental students perceive adverse effects or symptoms resulting from psychiatric medication use, and whether these effects negatively impact the clinical care provided by these students.

The high prevalence of mental disorders, affecting millions worldwide [5], and their consequences can lead to psychological distress, social isolation, and detrimental effects on human occupational activities [1]. Correspondingly, there has been an observed increase in the use of psychiatric medications [13, 14]. 

University students, including those in dentistry, experience elevated levels of stress and mental exhaustion, which have serious implications for their well-being and academic performance [20, 21]. A study conducted at the State University of Ponta Grossa in 2015 revealed that stress was a significant factor within the academic environment of the dentistry program, manifesting at various frequencies and intensities throughout the course of study [33]. Furthermore, a high pattern of drug use has been observed in Brazil, including among university populations, particularly stimulant psychotropic medications.[34–36].

Thus, the importance of collecting and analyzing information on this topic became evident. In this context, an electronic questionnaire was developed as a study tool due to its widespread use in data collection for research. This instrument allows for the systematic investigation of a specific population’s opinion on a particular subject, for which some prior knowledge already exists. In addition to being cost-effective, this method has the potential to reach a large number of respondents [37]. Literature review, besides being an effective method, is considered the primary resource for obtaining new assessment instruments [28]. Moreover, the use of existing instruments is recognized as a valuable resource in the development of new assessment tools [28]. 

Following the literature search, the questionnaire was organized into blocks according to the thematic areas addressed. Block 1 comprised socioeconomic questions (efamily income, age, ethnicity, and gender), block 2 included questions related to the medications used by participants (medication name, duration of use, and observed effects) and block 3 contained questions regarding the participants’ self-perception of any possible interference, or lack thereof, in their clinical practice.

Langoski et al. (2015) demonstrated that 45% of first-year dental students exhibited stress symptoms, while 55% of fifth-year students presented stress symptoms consistent with the stages of alarm, resistance, mild exhaustion, and exhaustion [33]. Based on this, the study population was limited to students in their final year, as they are better positioned to identify whether medication use was related to circumstances associated with the dental school environment, having accumulated greater academic experience compared to students in earlier years [33]. 

To develop the first block of questions, the Basic Questionnaire of the 2022 Demographic Census by IBGE (Brazilian Institute of Geography and Statistics) and the questionnaire used in the study titled “Assessment of Psychotropic Drug Use among University Students” were used as references for the formulation of some items [38]. Additionally, questions not present in these questionnaires were included.

The block 1 consisted of dichotomous questions (“yes” or “no”), single-choice objective questions, and open-ended questions. The Block 2, which focused on questions related to medications used by participants, was based in part on questionnaires from other studies [38, 39]. Furthermore, this block included questions that were not adapted from prior questionnaires. The block comprised objective items with single and multiple-choice responses with open alternatives, as well as one item with dichotomous alternatives (“yes” or “no”). Lastly, block 3, addressing participants’ self-perception regarding possible interference in clinical practice, was constructed with questions developed by the researchers, as no existing questionnaire was found addressing this specific objective. For the elaboration of Block 3, a numeric scale combined with a visual scale was employed, alongside a Likert frequency scale ranging from “never” to “very frequently.” Likert-type scales are widely used in research to measure respondent characteristics [40]. 

After the initial construction of the instrument, it underwent evaluation by a committee of experts. Content validation by a specialist committee is a critical step in construct validation and should include between five and ten members [26]. In the present study, the committee was composed of eight members, and the instrument was submitted to evaluation and re-evaluation until all items were approved following both quantitative and qualitative analysis. To complement the CVI analysis, the modified Kappa statistic was used for each item in the questionnaire, to minimize the chances of casual agreement and increase consistency in the experts’ assessment [31]. Thus, the agreement among experts was considered relevant to provide evidence of content validity for the instrument.

The final stage of construct validation involved administering the instrument to a pilot group. Pre-testing enables the identification of measurement errors within the instrument that could compromise the interpretation of scores. Moreover, it facilitates planning for a reliability study that includes these errors, allowing assessment [41]. This supports the use of pre-testing as a fundamental step in the development of the instrument employed in this study and certifies the research outcome. Upon completion of this stage and after adjusting two items, the final version of the instrument was produced.

It is important to acknowledge certain limitations of the present study. The World Health Organization (2014) [42] emphasizes that mental health encompasses functioning, autonomy, social inclusion, and the influence of structural determinants such as inequality, discrimination, and access to care, dimensions that were not addressed in the current questionnaire. Future studies may consider adapting the instrument to incorporate these psychosocial and contextual aspects.

## Conclusion

The methodology and research conducted in this study resulted in the development of a questionnaire capable of effectively assessing the prevalence of psychiatric medication use among dental students in Brazilian universities, as well as their perception of associated adverse effects or symptoms. Additionally, the instrument evaluates the potential negative impact of these effects on the clinical practice of the students. The developed questionnaire presents evidence of content validity and holds promise for application in future research endeavors.

## Supplementary Information

Supplementary Material 1.

## Data Availability

Data is provided within the manuscript or supplementary information files.
