# Supplementary material for: Development and content validation of an instrument to assess the prevalence of psychiatric medication use among dental students at Brazilian universities
Source: BMC Med Educ. 2025 Sep 10;25:1249. doi: 10.1186/s12909-025-07843-y (PMC12421737; doi:10.1186/s12909-025-07843-y)
Supplement: Supplementary file 1 — Supplementary Material 1. [file 12909_2025_7843_MOESM1_ESM.pdf]

QUESTIONÁRIO DA PESQUISA INTITULADA - PREVALÊNCIA DO USO DE MEDICAÇÕES PSIQUIÁTRICAS POR DISCENTES DE GRADUAÇÃO EM ODONTOLOGIA DE UNIVERSIDADES BRASILEIRAS.

Estamos convidando você a responder, anonimamente (seu nome e e-mail NÃO serão solicitados) e voluntariamente, este questionário cujo objetivo é avaliar a prevalência do uso de medicações psiquiátricas por alunos de graduação em odontologia de universidades brasileiras.
O tempo de preenchimento é de aproximadamente 06 minutos.
Ao responder o questionário, considere medicações psiquiátricas, psicotrópicos e psicofármacos como sinônimos.

Agradecemos sua inestimável colaboração.

1. A maioria das disciplinas que você está cursando faz parte da grade curricular do último ano da faculdade de odontologia em que você está matriculado?
Resposta única

( ) Sim.
( ) Não.

2. Em qual estado brasileiro está localizada a faculdade de odontologia que você estuda?
Resposta única

( ) Acre.
( ) Alagoas.
( ) Amapá.
( ) Amazonas.
( ) Bahia.
( ) Ceará.
( ) Distrito Federal.
( ) Espírito Santo.
( ) Goiás.
( ) Maranhão.
( ) Mato Grosso.
( ) Mato Grosso do Sul.
( ) Minas Gerais.
( ) Pará.
( ) Paraíba.
( ) Paraná.
( ) Pernambuco.
( ) Piauí.
( ) Rio de Janeiro.
( ) Rio Grande do Norte.
( ) Rio grande do Sul.
( ) Rondônia.
( ) Roraima.
( ) Santa Catarina.
( ) São Paulo.
( ) Sergipe.
( ) Tocantins.

3. A faculdade de Odontologia na qual você estuda é pública ou privada?
Resposta única

( ) Pública.
( ) Privada.

4. De acordo com a Sociedade Americana de Psicologia:
Cisgênero – é um indivíduo cuja identidade de gênero se alinha com seu sexo atribuído no nascimento.
Transgênero - pessoa cuja identidade de gênero e/ou papel de gênero não está de acordo com o que é tipicamente associado ao seu sexo atribuído no nascimento.
Não binário – gênero que é visto como algo entre ou além do gênero "binário" de homem e mulher, bem como gênero que incorpora elementos de homem e mulher.

Qual é a sua orientação de gênero?
Resposta única

( ) Cisgênero feminino.
( ) Transgênero feminino.
( ) Cisgênero masculino.
( ) Transgênero masculino.
( ) Não binário.
( ) Outro. ____________________.

5. Você se considera parte da comunidade LGBTQIAPN+ (Lésbicas, Gays, Bissexuais, Transgêneros, Queer, Intersexuais, Assexuais/Arromânticas, Pansexuais, Não-binárias e mais)?
Resposta única

( ) Sim, me considero.
( ) Não, não me considero.

6. Qual é a sua idade?
Resposta aberta (apenas números)

_______________ anos.

7. Qual é a sua cor/raça?
Resposta única

( ) Branca.
( ) Preta.
( ) Amarela.
( ) Parda.
( ) Indígena.

8. Quantas pessoas moram no mesmo domicílio que você? (Responda 0 – zero - se você mora sozinho).
Resposta aberta

__________ pessoa (s).

9. Qual é a faixa de rendimento bruto mensal da sua residência/ família? (Considere todas as pessoas que moram na residência. Considere o salário mínimo no valor de R$ 1.412,00)
Resposta única

( ) R$ 1,00 a R$ 500,00.
( ) R$ 501,00 a R$ 1.000,00.
( ) R$ 1.001,00 a R$ 2.000,00.
( ) R$ 2.001,00 a R$ 3.000,00.
( ) R$ 3.001,00 a R$ 5.000,00.
( ) R$ 5.001,00 a R$ 10.000,00.
( ) R$ 10.001,00 a R$ 20.000,00.
( ) R$ 20.001,00 a R$ 100.000.
( ) R$ 100.001 ou mais.

10. Quantos filhos (as) você tem?
(Responda 0 – zero - se você não tem filhos)
Resposta aberta

( ) 0 filho.
( ) 1 filho.
( ) 2 filhos.
( ) 3 filhos.
( ) 4 filhos.
( ) 5 filhos.
( ) 6 filhos.
( ) 7 filhos.
( ) 8 filhos.
( ) 9 filhos.
( ) 10 filhos.

Para responder às próximas questões considere que psicofármacos são substâncias químicas que agem no sistema nervoso central, atuando sobre a função psicológica e o estado mental, alterando, consequentemente, o comportamento, o humor e a cognição.
São incluídos os medicamentos com ações antidepressiva, ansiolítica, psicoestimulante, antipsicótica e/ou sedativa.
Ademais, considere como sinônimos os termos: psicofármaco, psicotrópico e medicação psiquiátrica.

11. Você usa ou já usou psicofármaco/ psicotrópico/ medicação psiquiátrica? (Mesmo que apenas uma vez)
Resposta única

( ) Sim, faço uso.
( ) Sim, já fiz uso.
( ) Não, nunca usei.

12. Quando foi o início da administração do psicofármaco/ psicotrópico/ medicação psiquiátrica?
Resposta única

( ) Antes do ingresso na Faculdade de Odontologia.
( ) Após o ingresso na Faculdade de Odontologia.

13. Qual é/foi a sua frequência de uso do psicofármaco/ psicotrópico/ medicação psiquiátrica?
Resposta única

( ) Utilizei uma única vez.
( ) Uso ocasionalmente (apenas em alguns dias. Não faço uso contínuo.)
( ) Faço uso contínuo.
( ) Fiz uso contínuo, mas parei.
( ) Nunca usei medicação psiquiátrica.

14. Quem foi o profissional prescritor do psicofármaco/ psicotrópico/ medicação psiquiátrica?
Resposta única

( ) Nenhum (automedicação).
( ) Psiquiatra.
( ) Clínico geral.
( ) Neurologista.
( ) Outro________________.

15. No caso de automedicação: qual(is) foi(foram) a(s) razão(ões) para o uso?
Resposta múltipla

( ) Ansiedade.
( ) Tristeza.
( ) Insônia.
( ) Desâmino.
( ) TPM.
( ) Dificuldade em se concentrar.
( ) Alteração do apetite.
( ) Alteração da libido.
( ) Alteração do peso.
( ) Mudança de comportamento.
( ) Irritação.
( ) Agressividade.
( ) Medo.
( ) Período de prova.
( ) Outra__________________.

16. No caso em que o psicofármaco/ psicotrópico/ medicação psiquiátrica tenha sido prescrito por profissional: qual(is) foi(foram) a(s) indicação(ões) clínica(s)?
Resposta múltipla

( ) Depressão.
( ) Transtorno de ansiedade.
( ) Transtorno de desenvolvimento.
( ) Transtorno de personalidade.
( ) Transtorno bipolar.
( ) Transtorno disfórico pré-menstrual.
( ) Transtorno de déficit de atenção e hiperatividade (TDAH).
( ) Insônia.
( ) Outra__________________.

17. Já aumentou ou diminuiu a dose do psicofármaco/ psicotrópico/ medicação psiquiátrica sem consultar o profissional prescritor da medicação?
Resposta única

( ) Sim.
( ) Não.

18. Qual(is) classe(s) de psicofármaco/ psicotrópico/ medicação psiquiátrica você utiliza/ utilizou?
Resposta múltipla

( ) ANTIDEPRESSIVOS (ex: amitriptilina/ amytril®, amoxapina, bupropiona/ zetron®, citalopram/ cipramil®, clomipramina/ anafranil®, desipramina/ norpramin®, dosulepina/ protiadene®, doxepina/ aponal®, escitalopram/ lexapro®, fenelzina/ nardil®, fluoxetina/ prozac®, fluvoxamina/ luvox®, imipramina/ tofranil®, isocarboxazida, lofepramina, maprotilina/ ludiomil®, mirtazapina/ remeron soltab®, moclobemida/ aurorix®, nefazodona, nortriptilina/ pamelor®, paroxetina/ aropax®, sertralina/ zoloft®, tranilcipromina, trazodona/ donaren®, trimipramina, triptofano, venlafaxina/ zyvifax®).
( ) ANSIOLÍTICOS (ex: buspirona/ buspar®, clorazepato, clordiazepóxido/ limbitrol®, clonazepam/ rivotril®, clonidina/ clonidin®, diazepam/ valium®, droperidol, halazepam, hidroxizina/ prurizin®, lorazepam/ lorax®, meprobamato, oxazepam/ serenal®, potássio, pregabalina/ lyrica®, propranolol/ inderal®).
( ) PSICOESTIMULANTES. (ex: anfetamina/ anfepramona®, atomoxetina/ strattera®, dextroanfetamina/ venvanse®, metanfetamina, metilfenidato/ ritalina®, pemolina/ Sectral®).
( ) ANTIPSICÓTICOS (ex: acetofenazina, amissulprida/ socian® , aripiprazol/ abilify®, clorpromazina/ amplictil®, clorprotixeno, clozapina/ leponex®, flupentixol/ fluanxol retard®, flufenazina/ flufenan®, haloperidol/ haldol®, levomepromazina/ neozine®, loxapina, mesoridazina, molindona, olanzapina/ zyprexa®, periciazina/ neuleptil®, perfenazina, pimozida/ orap®, proclorperazina, promazina, quetiapina/ seroquel®, risperidona/ risperdal®, sulpirida/ equilid®, tioridazina/ unitidazin®, tiotixeno/ navane®, trifluoperazina/ stelazine®, triflupromazina, zotepina).
( ) HIPNÓTICOS/ SEDATIVOS. (ex: alprazolam/ frontal®, difenidramina/ difenidrin®, estazolam/ noctal®, etclorvinol, flurazepam/ dalmadorm®, glutetimida, lormetazepam, nitrazepam/ sonebon®, pentobarbital/ nembutal®, prometazina/ fernegan®, propofol/ diprivan®, quazepam, secobarbital/ seconal®, temazepam/ normison®, triazolam/ halcion®, zaleplon, zolpidem/ stilnox®, zopiclona/ crestor®).
( ) ESTABILIZADORES DE HUMOR. (ex: carbamazepina/ tegretol®, gabapentina/ neurontin®, lamotrigina/ lamictal®, sal de lítio/ carbolitium®, valproato/ depakene®).
( ) outro________________________________.

19. Sente/sentiu algum efeito/sintoma indesejado decorrente do uso do psicofármaco/ psicotrópico/ medicação psiquiátrica?
Resposta única

( ) Sim.
( ) Não.

20. Marque os efeitos/sintomas indesejados que você sente/ sentiu decorrente do uso do psicofármaco/ psicotrópico/ medicação psiquiátrica.

Agitação: ( ) Sim ( ) Não
Resposta única

Alterações menstruais( ) Sim ( ) Não
Resposta única

Alterações visuais (por exemplo, visão turva): ( ) Sim ( ) Não
Resposta única

Ansiedade: ( ) Sim ( ) Não
Resposta única

Astenia (diminuição de força muscular): ( ) Sim ( ) Não
Resposta única

Aumento ou redução de libido: ( ) Sim ( ) Não
Resposta única

Aumento ou redução de peso: ( ) Sim ( ) Não
Resposta única

Confusão mental: ( ) Sim ( ) Não
Resposta única

Constipação (conhecido como prisão de ventre ou instentino preso, é caracterizada pela dificuldade constante ou eventual de eliminação das fezes):
( ) Sim ( ) Não
Resposta única

Comprometimento de memória: ( ) Sim ( ) Não
Resposta única

Comprometimento do controle motor: ( ) Sim ( ) Não
Resposta única

Diaforese (suor excessivo): ( ) Sim ( ) Não
Resposta única

Diarréia: ( ) Sim ( ) Não
Resposta única

Discinesia tardia (movimentos involuntários repetitivos): ( ) Sim ( ) Não
Resposta única

Dor de cabeça: ( ) Sim ( ) Não
Resposta única

Hipotensão ortostática (forma de pressão arterial baixa que gera tontura ao levantar-se da posição sentada ou deitada): ( ) Sim ( ) Não
Resposta única

Insônia: ( ) Sim ( ) Não
Resposta única

Irritabilidade: ( ) Sim ( ) Não
Resposta única

Queda de cabelo: ( ) Sim ( ) Não
Resposta única

Reações na pele: ( ) Sim ( ) Não
Resposta única

Sonolência: ( ) Sim ( ) Não
Resposta única

Taquicardia (aceleração batimentos cardíacos): ( ) Sim ( ) Não
Resposta única

Tontura: ( ) Sim ( ) Não
Resposta única

Vômito/Náusea: ( ) Sim ( ) Não
Resposta única

Outro(s)_______________.
Resposta aberta.

21. Em algum momento os efeitos colaterais apresentados te levaram a interromper o uso do psicofármaco/ psicotrópico/ medicação psiquiátrica?
Resposta única

( ) Sim.
( ) Não.

22. Quanto o uso da medicação psiquiátrica /psicofármaco/ psicotrópico interfere/ interferiu negativamente nas suas habilidades durante a prática clínica na faculdade de odontologia?

Considere:
10 – Interferência negativa máxima. Não consigo/consegui desenvolver a prática clínica na faculdade de odontologia.
0 – Não interfere/interferiu negativamente em nada durante a prática clínica na faculdade de odontologia.
Resposta única

( ) 10.
( ) 9.
( ) 8.
( ) 7.
( ) 6.
( ) 5.
( ) 4.
( ) 3.
( ) 2.
( ) 1.
( ) 0.

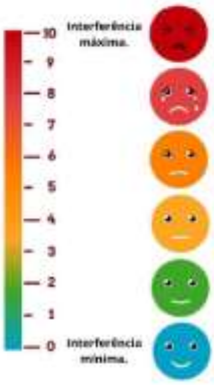

23. Marque a frequência na qual o uso do psicofármaco/ psicotrópico/ medicação psiquiátrica interfere/interferiu negativamente nas suas habilidades durante a prática clínica na faculdade de odontologia.

Eu sinto/senti efeitos indesejados do uso de psicofármacos durante o atendimento do meu paciente na faculdade de odontologia.
( ) Muito frequente
( ) Frequentemente
( ) Eventualmente
( ) Raramente
( ) Nunca
Resposta única

Na faculdade de odontologia, durante a consulta, algum paciente já percebeu efeitos indesejados decorrentes de psicofármacos que estou usando
( ) Muito frequente
( ) Frequentemente
( ) Eventualmente
( ) Raramente
( ) Nunca
Resposta única

Na faculdade de odontologia, durante o atendimento clínico, algum professor já percebeu efeitos indesejados decorrente de psicofármacos que estou usando.
( ) Muito frequente
( ) Frequentemente
( ) Eventualmente
( ) Raramente
( ) Nunca
Resposta única

Eu já precisei desmarcar uma consulta com meu paciente na clínica da faculdade de odontologia devido aos efeitos indesejados decorrente de psicofármacos que estou usando.
( ) Muito frequente
( ) Frequentemente
( ) Eventualmente
( ) Raramente
( ) Nunca
Resposta única

Os efeitos indesejados psicofármacos que estou usando, me levaram a causar um acidente/iatrogenia.
( ) Muito frequente
( ) Frequentemente
( ) Eventualmente
( ) Raramente
( ) Nunca
Resposta única
